# Rare and Insidious Toxicities from New Combination Therapies in Metastatic Renal Cell Cancer: Lessons Learned from Real-Practice

**DOI:** 10.3390/curroncol29100533

**Published:** 2022-09-22

**Authors:** Arianna Dri, Silvio Ken Garattini, Marika Cinausero, Marianna Macerelli, Martina Fanelli, Fabio Puglisi, Gianpiero Fasola, Paola Ermacora

**Affiliations:** 1 Department of Medicine (DAME), University of Udine, 33100 Udine, Italy; 2 Department of Medical Oncology, Azienda Sanitaria Universitaria Friuli Centrale (ASUFC), 33100 Udine, Italy; 3 Department of Medical Oncology, Centro di Riferimento Oncologico (CRO), IRCSS, 33081 Aviano, Italy

**Keywords:** renal cell cancer, multidisciplinary team, immunotherapy, targeted therapy, toxicities

## Abstract

The advent of immune checkpoint inhibitors in combination with multitarget tyrosine kinase inhibitors has become a standard first-line treatment for metastatic renal cell cancer. Along with survival improvement, new toxicities have emerged. Such adverse events are still complex to be managed and some of them are rare and could be insidious or even fatal. Medical oncologists dispose of guidelines about the management of toxicities from immune checkpoint inhibitors but not for combinations. Therefore, it is still difficult to properly attribute and manage additive or overlapping adverse events. We report two clinical cases regarding rare treatment-related endocrine toxicities—hypophysitis and thyroiditis—with particular focus on their management. To this purpose, immune checkpoint-related toxicities guidelines represent the starting point. However, their implementation with additional measures is needed, considering the increasing complexity of current clinical scenarios. The goal is to correctly recognize adverse events and address side effects, so as not to discontinue effective treatments. We, therefore, aim at discussing the points of proper management of toxicities and individuating potential areas of improvement.

## 1. Introduction

The combination of an immune checkpoint inhibitor (ICI) and a multi-targeted tyrosine kinase inhibitor (TKI) is a widely used standard first-line treatment for metastatic renal cell cancer (RCC). Based on the phase III trials conducted so far, the available association strategies are pembrolizumab plus axitinib (KEYNOTE 426), avelumab plus axitinib (JAVELIN Renal 101), nivolumab plus cabozantinib (Check Mate 9ER), and pembrolizumab plus lenvatinib (CLEAR) [1,2,3,4]. Among the therapeutic doublets, the combination of ipilimumab plus nivolumab has to be mentioned too (Check Mate 214) [5]. Both immunotherapy and targeted therapy have their own spectrum of toxicities, among which some are common and others rarer. The most important international oncology scientific associations provided extensive guidelines concerning the management of immune-related adverse events (AEs) [6,7]. Clinicians have learned to easily identify the most frequent ones such as skin rash, colitis, hepatitis, pneumonitis, and thyroid impairment, but other less pathognomonic presentations (i.e., hypophysitis and adrenal insufficiency) should not be underestimated. Immune-related toxicities could manifest at different timepoints, ranging from a few weeks since treatment start until after its discontinuation. Symptomatic control can be achieved through increasing doses of steroid and immunosuppressors, according to AEs severity [8]. Conversely, the most common TKI-related AEs—such as hand-and-foot syndrome, diarrhea, and hypertension—have been widely experienced by oncologists over the last decade in the context of RRC treatment with sunitinib, pazopanib, and axitinib as an example [1,9]. They usually occur a few weeks after the initiation of therapy, are dose-dependent, and could be handled through treatment discontinuation and dose reduction. However, ICI and TKI-related AEs could often overlap, and it may be difficult to attribute them to one specific drug. Moreover, some clinical effects can be related to the underlying disease as well or be a manifestation of patients’ known comorbidities. Therefore, the management of such toxicities can present some pitfalls and the major risk is to prematurely discontinue an effective treatment. This manuscript aims to provide descriptions of two rare toxicities from immunotherapy and TKI combinations and to propose a summary of recommendations for AEs management. In particular, we report two meaningful real-practice clinical cases in which asthenia was the onset symptom. Being a nonspecific disturbance, it that can be triggered by multiple causes. The challenge is to understand its etiology, manage it, and resume treatment if possible, as long as disease control is confirmed over time.

## 2. Case Presentation

### 2.1. First Clinical Case

The first clinical case deals with a 71-year-old man who was diagnosed with left RCC in February 2012. At baseline, the patient did not present relevant comorbidities, nor did he take any specific chronic drug therapy. He initially underwent radical left nephrectomy. Nine years later, the disease relapsed with multiple lung recurrences. The prognosis was classifiable as a ‘favorable risk’ group according to International Metastatic RCC Database Consortium (IMDC). A first-line combination treatment with pembrolizumab 200 mg and axitinib 5 mg bid was started, after providing the patient with proper information about expected benefits and possible side effects. Reassessment with a computed tomography (CT) scan was performed after four cycles of treatment with evidence of partial response to lung disease. The patient tolerated the therapy well, without complaining of any significant symptoms. After the ninth cycle, he started suffering from hand-foot syndrome (HFS) and significant fatigue affecting daily activities, both of grade 2 (G2) according to Common Terminology Criteria for Adverse Events (CTCAE) version 5.0. Being skin disorders more typically related to TKIs and fatigue, a non-specific symptom, axitinib was precautional withheld. Nevertheless, two weeks later the patient went to the acute oncology clinic because of worsening fatigue, lack of appetite, nausea, and limbs’ weakness. At the clinical evaluation, hypotension was detected. Blood tests showed hyponatremia and decreased plasma osmolarity. The entire pituitary stimulus-dependent hormone panel was tested by dosing plasmatic levels of cortisol, adrenocorticotropic hormone (ACTH), luteinizing hormone (LH), follicle-stimulating hormone (FSH), 17-beta-estradiol, testosterone, prolactin, insulin-like growth factor-1 (IGF-1), thyroid-stimulating hormone (TSH), free triiodothyronine, (fT3) and free thyroxine (fT4). ACTH and cortisol values were under standard limits, while prolactin was increased. No other significant alterations emerged. Intravenous hydration with saline solution was immediately started, in order to gradually restore sodium levels and blood pressure, so that the patient could return home safely.

**Figure 1:**
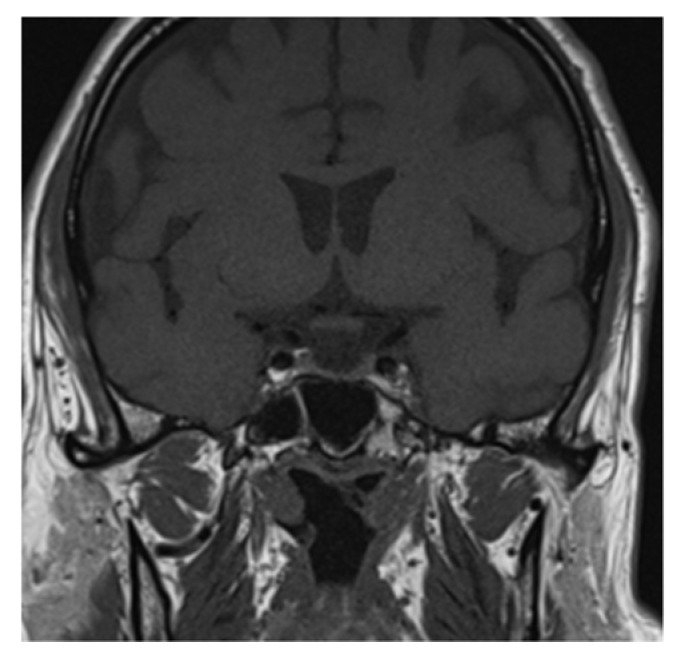
Partial “empty sellae syndrome” on brain MRI sagittal section. Pituitary gland reduced in size and lying along the floor of sphenoid sellae.

**Figure 2:**
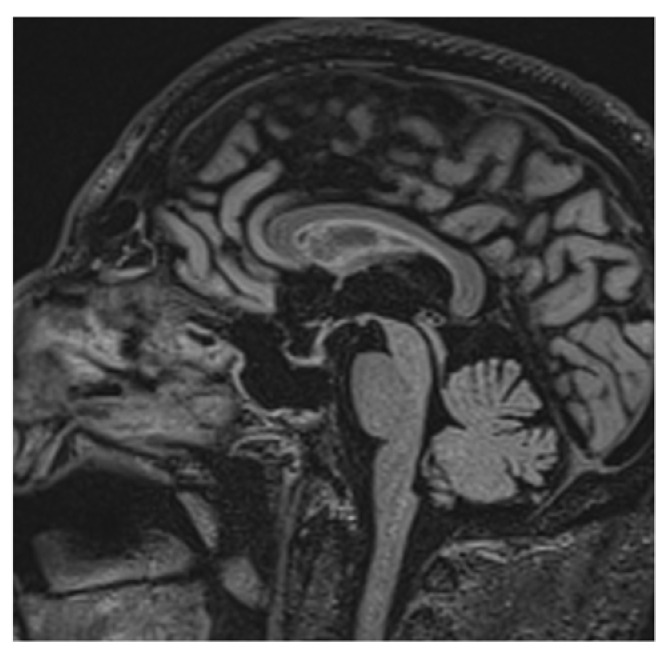
Partial “empty sellae syndrome” on brain MRI coronal section.

Clinical and laboratory findings were overall highly suggestive for immunotherapy-related adrenal insufficiency. Thereby, also pembrolizumab has been withheld and a low-dose steroid (dexamethasone 8 mg daily) was started, since the AE was classifiable as G2. Liquid restriction to a maximum of one liter per day was prescribed in order to control electrolyte impairment. After three days, the symptoms already improved. Further investigation with brain magnetic resonance imaging (MRI) was performed and it revealed a pituitary gland reduced in size, thin, and lying along the floor of the sphenoid sellae, consistent with partial “empty sellae syndrome” (Figure 1 and Figure 2). This finding led to a definitive diagnosis of iatrogenic hypophysitis and so secondary adrenal insufficiency. An endocrinological examination was performed and specific hormonal replacement therapy (HRT) with cortone acetate 37.5 mg daily was prescribed.

**Figure 3:**
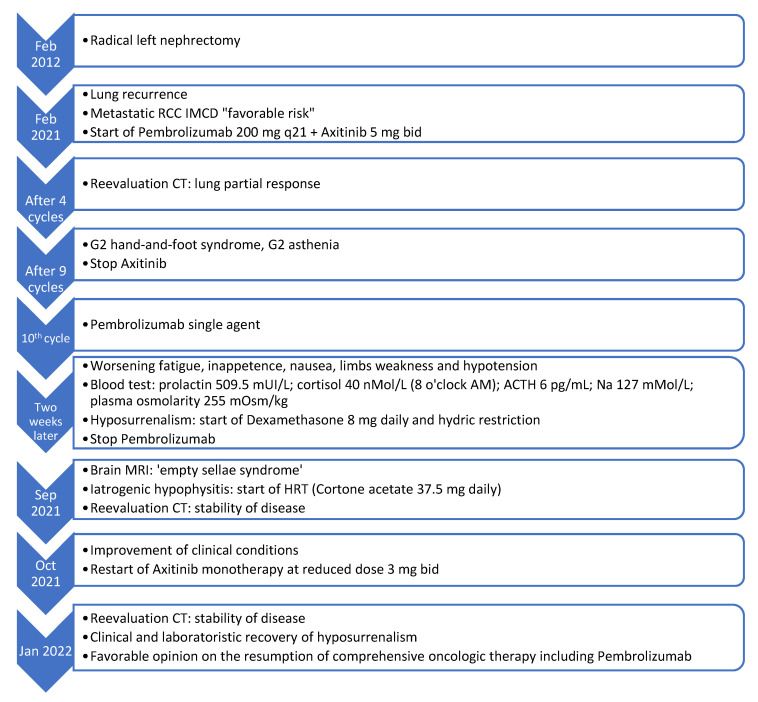
Events timeline of iatrogenic hypophysitis clinical case. RCC: renal cell cancer. IMDC: international metastatic database consortium. CT: computed tomography. AM: ante meridian. MRI: magnetic resonance imaging. ATCH: adrenocorticotropic hormone. G2: grade 2. HRT: hormonal replacement therapy. Normal ranges: TSH 0.4000–4.000 μUI/mL; fT3 2.30–4.20 pg/mL; fT4 8.90–17.60 pg/mL; prolactin 53–369 mUI/L; cortisol 150–650 nMol/L (8 o’clock AM); ACTH 6–49 pg/mL; Na 135–145 mMol/L; plasma osmolarity 275–295 mOsm/kg.

During HRT, the patient has been clinically and laboratory monitored, keeping hold of pembrolizumab. Once established that the symptoms could be attributed to ICI, axitinib has been resumed at a reduced dose of 3 mg bid, and monotherapy was continued for the next three months. Dose reduction was deemed necessary owing to the previous occurrence of severe hand-and-foot syndrome. A subsequent reassessment of a CT scan confirmed stable disease. Cortone acetate has been reduced to 25 mg daily considering clinical stability. The case has been re-examined within our hospital’s multidisciplinary board for immune toxicities and a team composed of medical oncologists and other specialists with expertise in the management of immunotherapy-derived AEs. The shared decision was to resume comprehensive oncologic therapy including pembrolizumab once clinical and laboratory resolution of adrenal insufficiency has been achieved, taking into account the proven effectiveness of the treatment. Figure 3 illustrates the temporal sequence of events that the patient experienced (Figure 3).

### 2.2. Second Clinical Case

**Figure 4:**
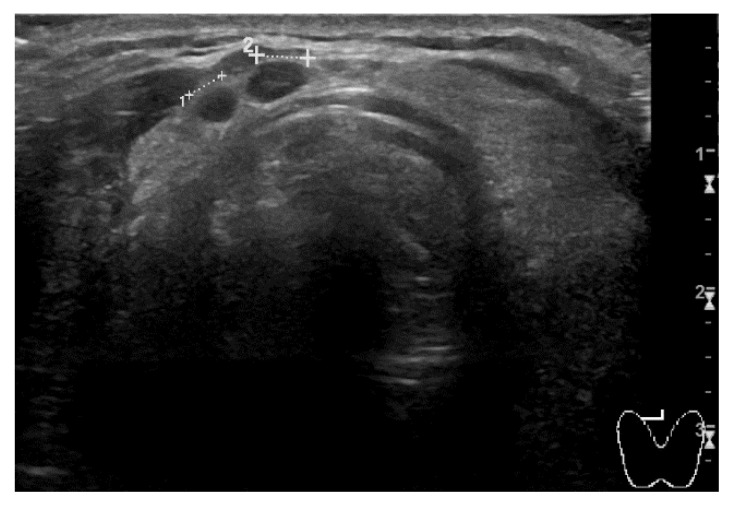
Immunotherapy-related thyroiditis on neck US examination. The thyroid gland is enlarged and swollen; uneven tissue pattern and nodular areas of phlogistic infiltrate are visible.

The second clinical case regards a man who was diagnosed with RCC of the left kidney. He first underwent partial nephrectomy in 2015. Two years later, radical nephrectomy was performed due to locoregional recurrence. At the follow-up CT scan of March 2021, right kidney and multiple lung metastases were detected. The prognosis was scored as a ‘favorable risk’ group according to the IMDC algorithm. The patient was 64-year-old at the time and his only significant comorbidities were hypertension and diabetes mellitus type II, both pharmacologically controlled by atenolol, linagliptin, and metformin. The patient was provided with comprehensive information and first-line therapy with three-weekly pembrolizumab 200 mg; axitinib 5 mg bid was promptly started. After two cycles of treatment, blood pressure values were no longer under control with the usual therapy, and the man started complaining about new onset G2 fatigue. Blood pressure control was rapidly achieved by adding amlodipine to the usual therapy and temporarily discontinuing axitinib for one week. Later, the patient noticed a left lateral-cervical swelling area of new appearance. A neck ultrasound (US) examination was performed, and it revealed multiple thyroid nodules consistent with toxic goiter (Figure 4). No thyroid hormone alterations were detected on blood tests, thus no specific action has been necessary.

At the end of the fifth cycle of treatment, the patient reported hearing impairment, progressive fatigue, and mood changes as he felt easily irritable. Axitinib has been withheld, and in-depth endocrinological examinations were performed by dosing plasmatic levels of TSH, fT3, fT4, LH, FSH, ACTH, and cortisol. The presence of an autoimmune component was investigated by testing anti-thyroglobulin, anti-thyroperoxidase, and anti-TSH receptor antibodies. Reduction of TSH, with concomitant elevation of fT3 and fT4 emerged. Autoimmune antibodies were absent. Endocrinological evaluation was performed, hence clinical and laboratory findings led to the diagnosis of immune-related thyrotoxicosis G2. Thyrostatic therapy with tapazole 5 mg bid was initiated. The heart rate was within standard limits, probably due to the beta-blocker the patient was already taking. A fine needle aspiration biopsy was not deemed necessary, as thyroid nodules represent a usual inflammation-associated outcome. A reassessment CT scan performed in July 2021 documented stability of lung disease and the partial response of right kidney lesion. A follow-up thyroid exam confirmed a radiological picture consisting of thyrotoxicosis, as the gland persisted enlarged and swollen.

**Figure 5:**
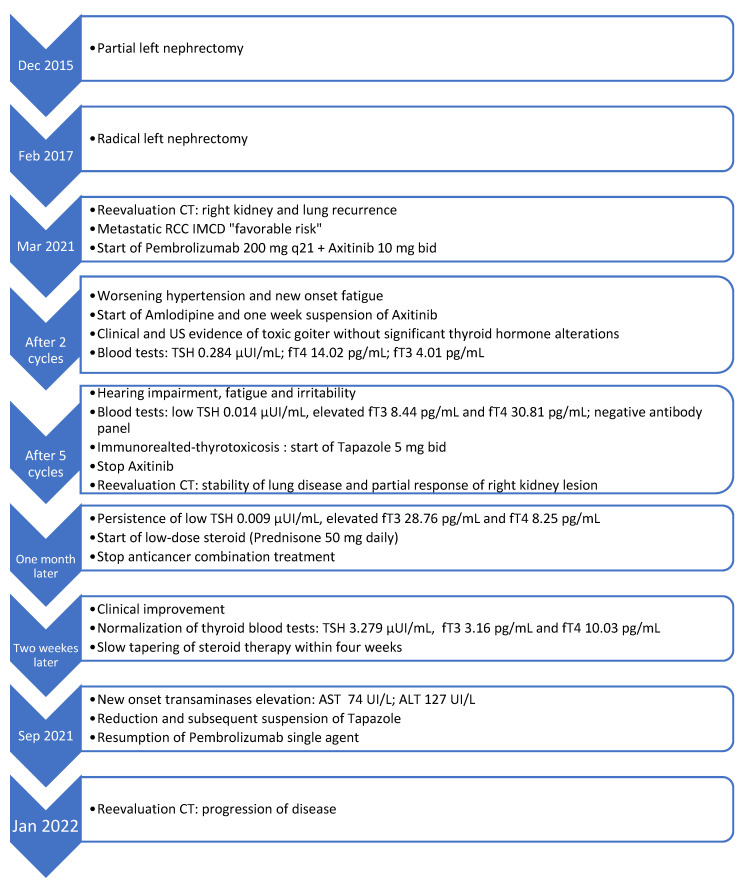
Events timeline of iatrogenic hyperthyroidism clinical case. RCC: renal cell cancer. IMDC: international metastatic database consortium. CT: computed tomography. US: ultrasound. ATCH: adrenocorticotropic hormone. fT3: free triiodothyronine. fT4: free thyroxine. G2: grade 2. AST: aspartate aminotransferase. ALT: alanine aminotransferase. Normal ranges: TSH 0.4000–4.000 μUI/mL; fT3 2.30–4.20 pg/mL; fT4 8.90–17.60 pg/mL; AST 4–40 UI/L; ALT 127 UI/L.

After four weeks of thyrostatic therapy, the patient no longer felt irritable but TSH value maintained low, while fT3 and fT4 persisted high. Thus, a low-dose steroid (prednisone 50 mg daily) was started and anticancer combination therapy has been kept suspended. Two weeks later, thyroid blood parameters returned to normal range, so steroid therapy was slowly tapered within four weeks. Meanwhile, an elevation of transaminases was detected during routine laboratory monitoring. The dosage of tapazole has been reduced to 5 mg on alternate days, being held accountable for the alteration; it was permanently discontinued at the beginning of September 2021. After recovery of thyroid function, anticancer treatment has been cautiously resumed with the pembrolizumab single-agent, according to endocrinological consultation without evidence of new immune-related toxicity. Unfortunately, restaging CT performed in January 2022 revealed progression of the disease, thus second-line treatment with cabozantinib was prescribed. Figure 5 illustrates the temporal sequence of events that the patient experienced (Figure 5).

## 3. Discussion

It is now well established that combination therapy with an ICI and a TKI provides better results in terms of response rates, progression-free survival, and overall survival when compared to the previous standard of care, namely sunitinib [10,11]. Currently available therapeutic association strategies include pembrolizumab plus axitinib, avelumab plus axitinib, nivolumab plus cabozantinib, and pembrolizumab plus lenvatinib, along with the immunotherapy doublet ipilimumab plus nivolumab [1,2,3,4,5]. To date, there are still no well-defined clinical criteria or predictive biomarkers that can guide the selection of the best first-line treatment. No direct comparisons among the various options have been performed up to now. However, updated indirect analyses including the aforementioned registration trials have been conducted. Focusing on toxicities and treatment tolerability, one found that the combination of lenvatinib plus pembrolizumab resulted associated with higher rates of treatment-related grade ≥3 AEs and treatment discontinuation [10]. Another systematic review and network metanalysis pointed out that only nivolumab plus ipilimumab seems to be associated with a lower likelihood of AEs compared to sunitinib. Among ICI/TKI combinations, the likelihood of providing the lowest grade 3 AEs is higher for pembrolizumab plus axitinib, followed by nivolumab plus cabozantinib, then lenvatinib plus pembrolizumab. It is important to note that the combination of two ICIs could cause toxicities protracted in time, despite fewer grade 3 events. To the contrary, the TKI-ICI combination more often causes AEs that easily resolve upon discontinuation of the targeted molecule [12]. Moreover, it should be recognized that it is hard to make an objective comparison between the side effects of these combination therapies since very often their safety profile does not match that of the respective single agent treatments. Toxicities are the result of two molecules acting synergistically and it is often difficult to attribute them to a specific one [11]. Indirectly derived data would need perspective confirmation through comparative trials. In fact, some trial results are still immature, and the characteristics of enrolled patients are not homogeneous. Clinicians should choose the most reasonable option taking into account personal experience with a certain regimen, the overall patient’s performance status and comorbidities, tolerability, prognostic class risk, and disease characteristics [10,11]. Moreover, it must be considered that regulatory approval status is not the same worldwide.

The most frequent toxicities recorded with pembrolizumab and axitinib combination are diarrhea, hypertension, fatigue, and hypothyroidism. Anyway, rarer AEs should not be underestimated. Endocrine toxicities are predominantly immune-related, along with several further side effects that can interest other systems such as gastrointestinal and osteoarticular apparatus. TKIs are usually related to a slightly different spectrum of AEs, being the most typical hypertension, HFS, cardiovascular disorders, and hepatotoxicity [1]. Nonetheless, onset symptoms of ICIs and TKIs toxicities could often be non-specific (i.e., nausea, vomiting, loss of appetite, tiredness, weakness, temporary hair loss, cough, headache, altered sense of taste, hoarseness, and diarrhea or constipation), therefore the responsible medication results are difficult to identify. Clinical management is made more challenging by the fact that the symptoms could also be attributable to neoplastic disease or patients’ anamnestic comorbidities, therefore multidisciplinary board discussion is often needed [13].

Focusing on endocrine toxicities, hypophysitis is defined as an inflammatory condition of the anterior lobe of the pituitary gland and it configures as a rare immune-related AE. It is more commonly associated with CTLA-4 inhibitors. In particular, the incidence of hypophysitis can be estimated as 6.4% with a combination treatment of ipilimumab and nivolumab and about 0.5% with single-agent, anti-PD-1/PD-L1 [14]. Its pathogenesis is not yet fully understood. The median time of onset is between three and nine months from the beginning of the treatment. It can clinically present with mass effect symptoms, such as headache and visual impairment. Adrenal insufficiency symptoms such as fatigue, hypotension, mood alteration, anorexia, nausea, and vomiting could also occur. Sometimes patients complain about sexual disorders due to low testosterone levels. Neurological disturbances require immediate evaluation, as differential diagnosis includes encephalic or leptomeningeal neoplastic involvement and cerebrovascular acute events. Very often the first sign of toxicity emerges from blood routine tests, which can highlight low levels of pituitary hormones such as TSH, ACTH, FSH, and LH, or electrolytic disorders such as hyponatremia and hypokalemia. Brain MRI can help in doubtful situations, as the pituitary gland appears swollen or enlarged in an inflammatory phase, as well as shrunken in chronic damage. Hyperthyroidism is a very rare immuno-related AE and manifests in about 0.6–3.2% of patients treated with ICIs. It frequently represents a transient disorder that anticipates hypothyroidism. In fact, thyroid dysfunction typically has a biphasic course. Hyperthyroidism on average occurs three months after the beginning of the treatment. Alarm symptoms include alteration of the mood—jitteriness, agitation, mental confusion-, tachycardia, fever, and diarrhea. Blood tests are crucial for the diagnostic work-up as low TSH and elevated values of fT3 and fT4 are pathognomonic. Clinicians are used to recognizing and managing hypothyroidism, being it is very frequent, while the proper management of hyperthyroidism is often less obvious to medical oncologists due to its rarity [6,14].

As per international guidelines, the accurate diagnosis of endocrine toxicities should not be delayed, and rapid appropriate action must be taken. When immunotherapy is held responsible for the AEs, subsequent management is clearly explained in the main oncologic guidelines and it varies according to the severity. Mild symptoms or alterations only detectable by blood tests are classifiable as G1 AEs. In these cases, ICI can be continued under close monitoring. Supportive therapy should be started only if necessary. Suspension of the treatment can be considered if disturbances significantly affect patients’ quality of life. In the case of G2 AEs, ICI should be promptly stopped and treatment consisting of HRT should be started if needed. Low-dose steroid therapy (prednisone 0.5–1 mg/kg/day) should be considered. Immunotherapy can be resumed if toxicity resolves or at least returns to grade 1. If serious neurological impairment occurs, a high-dose steroid should be given. When a grade 3 (G3) adverse event manifests, ICI must be discontinued and high-dose steroid therapy must be started (1–2 mg/kg/day), while continuing to closely monitor patients’ clinical condition. If toxicities improve within a few days, a full dose steroid could be maintained as long as deemed necessary, then a slow tapering should be performed over at least 4–6 weeks. On the contrary, immunosuppressive therapy should be implemented considering the addition of other immunosuppressive agents such as infliximab or mycophenolate mofetil for colitis or hepatitis. Grade 4 (G4) toxicity compels permanent discontinuation of ICIs [6,7]. Generally, long-term HRT is required in most patients even after passing through the acute phase regardless of toxicity degree. Immunotherapy can often be resumed upon tapering to the maximum allowed dose of steroid (prednisone 10 mg daily).

Management of side effects could be more challenging in the case of combination therapy with a TKI and an ICIs. A very useful tool for managing axitinib/ICI combination toxicities is the consensus article by Grunwald and colleagues. According to this article, G1 fatigue, hepatitis, and diarrhea toxicities should be managed by close clinical monitoring over time and continuation of full-dose oncology treatment. In case of G2 AEs, axitinib only should be initially discontinued. If clinical improvement is achieved within a few days (48–72 h), it probably means that TKI was the causative agent. Thus, axitinib treatment can be adjusted through dose reduction. On the contrary, in the absence of clinical improvement, ICIs administration must be withheld while starting low-dose steroid treatment. If these measures lead to symptoms’ resolution, then restarting axitinib could be considered, followed by ICI rechallenge. For higher severity AEs, such as G3 and G4, both molecules must be immediately suspended. The patient must undergo specialist’s clinical examination and hospitalization should be considered whether needed. If appropriate supportive therapy allows clinical improvement at least at G1, restarting of axitinib may be considered for G3 toxicities, followed by ICI rechallenge. Contrarily G4 AEs require permanent suspension of ICI, while TKI monotherapy could be restarted [13].

In both reported clinical cases, the discontinuation of the axitinib was the first step, as it was considered the most likely responsible for the symptoms reported by patients. If the disturbances do not improve over a relatively short period of time, discontinuation of immunotherapy should also be considered. In the first case, it is peculiar that disorders appearing attributable to the TKI then turned out to be the telltale hint of a more insidious immune-related AE. It can be deduced that even supposedly trivial and common symptoms, such as asthenia, must not be underestimated in cancer patients. The etiology of such disturbances may lie in multifactorial causes, but treatment toxicity should always be included in differential diagnoses. In the case of clinical-radiological stability of disease resumption of oncologic therapy appears crucial if not prevented by an excessively high-grade AE. Since the available classes of drugs against renal cancer are still few, it is essential not to prematurely discontinue effective treatments. In this perspective, it is good practice to cautiously resume treatment with one drug first, even with reduced dose if deemed necessary and feasible, in order to assay tolerance and therefore restart full treatment.

It is partly true that oncology is becoming increasingly sectorial since nowadays high-patient load hospitals rely on teams of medical oncologists who are dedicated to the care of a specific discipline, of which the main ones are breast, lung, gastrointestinal, and genitourinary cancer. Skills-sharing is fundamental and recommended as many therapies are now transversal for most solid cancers. Guidelines represent the starting point for the management of treatment-related AEs, but the field is still challenging due to increasing clinical complexity. In this perspective, it would be necessary to go beyond the classical mono-pathology groups and to create multidisciplinary teams dedicated to emerging issues, such as an immunotoxicity multidisciplinary team. The team should be composed by experts in various clinical fields that the medical oncologist could rely on—including an endocrinologist, a rheumatologist, a gastroenterologist, a pneumologist, a neurologist, and a hepatologist. Simple handling AEs can be managed by the oncologists alone, while they should appeal to the multidisciplinary group in case of difficult challenges. Converging insidious clinical cases to dedicated specialists will help enrich each other’s expertise in toxicities management, so as to be successfully applied to upcoming situations. Case reports and clinical audits are also important to increase knowledge, as sharing personal experience in literature can lead to new standards. Knowledge transmission should be encouraged also among clinicians working in Emergency Department and in Territorial Care—as General Practitioners and Simultaneous Care Teams staff—in order to rapidly detect disturbances and facilitate patients’ access to proper specialist evaluations. Our cases confirm that a multidisciplinary approach including the involvement of dedicated specialists and fellow oncologists, is often critical for reassurance regarding the diagnostic-therapeutic management of insidious AEs. Sharing information has been crucial both to get advice on how to proceed and to make the personal experience available for future situations.

Patients themselves have an essential role in AEs management right from the start because they often first realize if there is any new onset symptom worthy of further investigation, as evidenced in reported cases. In fact, routine blood tests are not always helpful at the AEs onset and dedicated communication with the patients is the only element that clinicians can rely on. While offering an oncologic treatment, detailed information about expected benefits and potential related side effects must be provided, so that onset symptoms can be immediately identifiable. A recent online survey that explored patients’ preferences and expectations about systemic therapy in RCC found that patients rank efficacy as the most important outcome when considering treatment options. On the contrary, toxicity, time-off therapy, and cost are not significant priorities [15]. Moreover, Mansfield et al. proved that patients who are informed about the possible association between some of the AEs and PFS could be more adherent to therapy in presence of greater toxicity, if this would turn into better outcomes. The magnitude of association between AEs and the effectiveness of treatment has not been well established yet. Anyway, this project points out how comprehensive information about medication, including toxicities, efficacy, and their potential correlation, improves compliance and could optimize outcomes [16]. Nevertheless, patients should be properly informed and accurately warned not to overlook possible alarm bells because immunotherapy-related AEs may occur even after treatment discontinuation.

## 4. Conclusions

Most of the toxicities related to immunotherapy and TKIs are now known and easily manageable, others are infrequent but should not be overlooked as they can result in insidious or even fatal outcomes. Proper attribution of an AE to ICI or to TKI could be challenging, thus early discontinuation of an effective treatment is a concrete risk. Our case reports highlight how to successfully manage drugs’ combination of AEs through some focal points.

First of all, it results in essentially the adherence to international guidelines concerning immune-related toxicities and the application of proper predefined algorithms. The value of sharing becomes paramount since news drugs are increasingly appearing in the medical landscape in the last few years. Therefore, it is essential to gather the experiences of as many specialists as possible. The creation of multidisciplinary dedicated teams is a standard that major hospitals must pursue, in order to provide the best support in the pathway of care. Last but not least, the valuable role of patients should not be underestimated. It is crucial for clinicians to establish an effective relationship of trust with patients and to provide accurate information in order to make them feel an active part of the therapeutic process.

In conclusion, the integration of the guideline’s recommendations, along with knowledge-sharing and substantive involvement of patients, is the key to achieving optimal therapeutic management and the greatest benefits.

## Data Availability

All the data is contained within the manuscript.
